# Patterns of Migration Following Dementia Diagnosis

**DOI:** 10.1001/jamanetworkopen.2024.39499

**Published:** 2024-10-14

**Authors:** Momotazur Rahman, Bishnu Bahadur Thapa, Christopher Santostefano, Pedro Gozalo, Ulrike Muench, Cyrus M. Kosar, Hyesung Oh, Elizabeth White, Vincent Mor

**Affiliations:** 1 Department of Health Services Policy and Practice, Brown University, Providence, Rhode Island; 2 Department of Social & Behavioral Sciences, University of California, San Francisco, Sausalito; 3 Program of All-inclusive Care for the Elderly, Riverside, Rhode Island

## Abstract

**Importance:**

Diseases have historically prompted individual relocations to mitigate the risk of disease acquisition or improve access to care. As dementia prevalence increases, comprehending the migration patterns of affected individuals is vital for public policy.

**Objective:**

To quantify the association of dementia diagnosis with migration patterns by examining the proportion of individuals with dementia who relocate, the timing of their moves relative to diagnosis, and the nature of their new living arrangements, whether in institutional settings or different households.

**Design, Setting, and Participants:**

This cohort study leveraged a comprehensive dataset of national Medicare claims and assessments spanning from 2012 to 2020, including Medicare Beneficiary Summary File and nursing home administrative datasets. The study focused on beneficiaries who received diagnoses in 2016 of dementia, myocardial infarction, chronic obstructive pulmonary disease, or colon cancer. Analyses were performed from March 2023 to August 2024.

**Main Outcomes and Measures:**

The primary outcome was migration, defined as change in county or state. The analysis distinguished between migrations with and without a nursing home stay. By tracking patients’ residential county for 4 years before and after diagnosis, a difference-in-differences approach was used to contrast migration tendencies associated with dementia against the other 3 conditions.

**Results:**

The sample included 1 626 127 Medicare beneficiaries (mean [SD] age, 80.1 [8.0] years; 922 194 women [56.7%]) who received diagnoses of the 4 conditions in 2016. In total, 818 862 had a new dementia diagnosis (age, 82.0 [7.8] years; 492 146 women [60.1%]). Comparing between the prediagnosis and postdiagnosis months, the proportion migrating to a different county increased by 8.5 percentage points (95% CI, 7.6-9.4 percentage points) for individuals with dementia and between 4.2 to 5.8 percentage points among those with myocardial infarction, chronic obstructive pulmonary disease, or colon cancer. The difference-in-difference estimates indicated a 3.9 percentage point (95% CI, 3.7-4.0 percentage points) increase in intercounty migration and a 1.9 percentage point (95% CI, 1.8-2.0 percentage points) increase in interstate migration for patients with dementia, effectively doubling the likelihood of migration compared with the other conditions. Of the excess migrations resulting from dementia diagnosis, 55% occurred in community settings, and 45% occurred in institutional settings.

**Conclusions and Relevance:**

In this retrospective cohort study of Medicare fee-for-service beneficiaries, dementia was associated with a marked increase in migration rates over other major illnesses. This finding underscores the need to understand the factors associated with these distinct migration behaviors.

## Key Points

**Question:**

Is a dementia diagnosis associated with the likelihood and nature of migration compared with other major illnesses?

**Findings:**

In this cohort study of more than 1 million Medicare fee-for-service beneficiaries, individuals with a diagnosis of dementia were almost twice as likely to migrate to another county or state compared with those with diagnoses of myocardial infarction, chronic obstructive pulmonary disease, or colon cancer. Of the excess migrations resulting from dementia diagnosis, 55% occurred in community settings, and 45% occurred in institutional settings.

**Meaning:**

The marked increase in migration among patients following a dementia diagnosis highlights a distinctive need for policy and support interventions tailored to their unique migration patterns and care requirements.

## Teaser

This cohort study examines whether a diagnosis of dementia is associated with the likelihood of migration between counties or states compared with other major illnesses, including colon cancer, chronic obstructive pulmonary disease, and myocardial infarction.

## Introduction

Throughout history, diseases have exerted a profound influence on human migration patterns. In the face of major pandemics, such as bubonic plague, or more recent outbreaks like Ebola, the risk of infection has frequently served as a compelling reason for people to migrate.^1,2^ The search for improved access to health care and treatment, as exemplified by the HIV epidemic in the US and around the world, has also stimulated migration. However, to our knowledge, no one has explored the association between chronic disease incidence and migration. In modern times, high-income countries have undergone a demographic transition by which chronic, rather than infectious, diseases are the most prevalent. One of the most rapidly growing aging-related chronic conditions is dementia, a progressive neurological condition characterized by cognitive and functional decline that leads to impairment in the ability to perform activities of daily living.^3^ The global prevalence of dementia is estimated to increase by 166% from 2019 to 2050.^4^ In the US, the proportion of older adults dying with dementia increased from 35% in 2004 to 47% in 2017.^5,6^ As dementia progresses, individuals require more daily support. They may be compelled to migrate to be closer to family and other informal (unpaid) caregivers or to access formal (paid) long-term care services.

This study aims to document how a new dementia diagnosis is associated with migration. This study defines migration as relocating to a different county or state, a delineation informed by Medicare’s treatment of counties as distinct health care and insurance markets. Moving to a different county often means leaving behind familiar health care practitioners, friends, family, and other forms of community support. In addition, moving to a different state subjects individuals to different long-term care policies and Medicaid programs, further underscoring the importance of such a geographic transition. Thus, moving to a different county or state captures not only the administrative nuances of federal health care’s operational divisions but also encompasses broader shifts in service availability, economic landscapes, social support structures, and environmental factors, all of which can have profound impacts on the well-being and care choices of older adults. We used Medicare data to track address changes among Medicare fee-for-service enrollees with an incident dementia diagnosis in 2016 over 4 years before and after the diagnosis. As a comparison, we also investigated migration patterns for older adults with incident acute myocardial infarction (MI), chronic obstructive pulmonary disease (COPD), and colon cancer diagnoses during the same time window. We chose the 3 conditions for comparison because they, along with dementia, are common among older US individuals and represent major health events that require ongoing medical intervention and could potentially trigger migration.

## Methods

This cohort study was approved by the Brown University institutional review board, which waived the requirement for participant informed consent because the data were publicly available and deidentified, in accordance with 45 CFR §46. The study followed the Strengthening the Reporting of Observational Studies in Epidemiology (STROBE) reporting guidelines.

### Data Sources

We used the Medicare Beneficiary Summary File and nursing home administrative datasets to extract information on our study sample. The Medicare Beneficiary Summary File provides information on demographic characteristics, Medicaid enrollment, residential location, and chronic conditions for all Medicare beneficiaries. The Minimum Data Set is a standardized clinical assessment completed at regular intervals for all those using a Medicare and Medicaid–certified nursing home. This allows for the identification of nursing home residency. Using the Residential History File algorithm,^7^ we linked these data files to create a per-person chronological history of nursing home care utilization and location of residence. Our study window comprised 9 years, from January 1, 2012, to December 31, 2020, with the first 4 years serving as the prediagnosis period and the last 4 years as the postdiagnosis period.

### Study Population

We included Medicare fee-for-service beneficiaries who were enrolled in Medicare in 2012 and received new diagnoses in 2016 of 1 of 4 conditions: dementia, MI, COPD, or colon cancer. We classified beneficiaries as having new diagnoses using onset dates and incident flags for the following Chronic Condition Warehouse categories within the Master Beneficiary Summary File: Alzheimer disease and related dementias (ADRD), acute MI, COPD, and colon cancer. We restricted the analysis to beneficiaries with 48 months of observable data before diagnosis. We excluded Medicare Advantage enrollees because we cannot identify diagnoses and the onset dates. Approximately 6% of the individuals entered the sample more than once because they received incidental diagnoses of more than 1 of the 4 conditions in 2016. We allowed individuals to be present in more than 1 cohort under the assumption that acquiring other conditions, including the 4 we focus on, in 2016 or any follow-up years is a part of the aging process.

Our initial step involved the creation of a person-month level dataset encompassing a span of 97 months surrounding the diagnosis, 48 months preceding and 49 months following, with the month of diagnosis itself categorized as a postdiagnosis month. Every individual is consistently present for the full 48 months in the prediagnosis phase; however, the data for the postdiagnosis months vary according to the individual’s survival. To facilitate our multivariable analysis, we subsequently collapsed this dataset such that an individual was represented by 2 distinct observations corresponding to the periods before and after their diagnosis.

### Outcomes

Our outcome, migration to another county or state, is elucidated through county and state Federal Information Processing Standards codes, as recorded monthly in the Master Beneficiary Summary File. For migration on an individual-month level, we examined both incident and cumulative migration. Incident migration is defined as any change in county or state address relative to the previous month. Cumulative migration is defined as whether the person moved to another county or state in any of the earlier observation months.

With age, the likelihood of moving to a nursing home increases, generally leading to a change in residence. As a result, we also examined migration with or without a nursing home stay, defined as migration to a different county and/or state with or without a nursing home stay in the same month. For individual prediagnosis and postdiagnosis period analyses, we created binary indicators of any migration to a different county or state in a given period. We also examined the likelihood of moving to another place 15 miles or further as an outcome.

### Other Variables

We included age, race and ethnicity (American Indian, Asian, Black, Hispanic, and White) as extracted from the Master Beneficiary Summary File, sex, and Medicaid eligibility as reported on the month of diagnosis. Data on race and ethnicity were included to reduce the potential for confounding due to various socioeconomic factors associated with these constructs. We included comorbidities present at diagnosis derived from the Chronic Condition Warehouse’s 27 chronic condition categories. We grouped individuals into 3 categories according to the count of conditions: 0 to 7, 8 to 13, and 14 or more chronic conditions. We also included the metropolitan status of the residential county and the social deprivation index^8^ of the residential zip code on the basis of the individual’s first month of observation.

Because survival is a prerequisite for migration, we included months of survival following diagnosis in all of our analyses. We operationalized survival as a continuous measure, capped at 48 months, with 0 as the month of diagnosis.

### Statistical Analysis

Analyses were performed from March 2023 to August 2024. We first examined the summary statistics of the beneficiary characteristics for individuals with the 4 diagnoses. For the 97-month follow-up period between (48 months before to 49 months after diagnosis), we plotted the monthly proportion of the population with each diagnosis that moved to another county or state in a community setting or nursing home.

Because migration is a cumulative concept, we also plotted the cumulative probability of migration by months of follow-up (48 months before to 49 months after diagnosis). To assess how the difference in the likelihood of cumulative migration between the ADRD cohort and the rest of the 3 diagnosis cohorts combined changes with months of follow-up, we estimated a dynamic difference-in-differences model.^9^

As supplemental analyses, we plotted the likelihood of any migration to another county in a community setting and a nursing home in the entire 8 years with respect to the number of months survived following diagnosis, separately for individuals with ADRD and with the other 3 diagnoses. We also plotted the proportion of people who migrated to a place 15 miles or further away from the first observed zip code.

For the difference-in-difference analyses, we restructured the data so that each individual was represented with 2 distinct observations: one for prediagnosis and another for postdiagnosis. We calculated the proportion of individuals who migrated in the prediagnosis 48 months for individuals with different diagnosis groups. We then estimated the change in the proportion of individuals who migrated during the postdiagnosis period after adjusting for survival. To address potential nonlinearity and the truncated nature of the variable, we included the number of months an individual was observed in a specific period as a factor variable. Finally, to estimate excess migration due to dementia diagnosis compared with the other 3 diagnoses, we estimated difference-in-differences models comparing the postdiagnosis period with the prediagnosis period for the ADRD cohort and the other 3 diagnosis cohorts combined. These models are estimated as multivariable linear probability models, including individual fixed effects and the number of observed survival months fixed effects. The analytic file was built using SAS statistical software version 9 (SAS Institute), and all analyses were conducted in Stata statistical software version 17 (StataCorp). The 95% CIs are based on robust SEs. Statistical significance is defined as a 95% CI that does not cross the null value of 0.

## Results

Table 1 presents descriptive statistics for the different disease cohorts. The sample consisted of 1 626 127 fee-for-service Medicare beneficiaries (mean [SD] age, 80.1 [8.0] years; 922 194 women [56.7%]) who had new diagnoses of dementia, MI, COPD, or colon cancer captured in Medicare claims in 2016. The dementia group (818 862 individuals) was the oldest (mean [SD], 82.0 [7.8] years), and the COPD group was the youngest (mean [SD], 77.8 [7.6] years). The majority of individuals in each group were female, with the lowest proportion in the MI group (99 687 women [50.0%]) and the highest in the dementia group (492 146 women [60.1%]). Most individuals across all groups were White (1 426 476 individuals [87.7%]); 8225 individuals (0.5%) were American Indian, 26 887 (1.7%) were Asian, 138 377 (8.5%) were Black, and 26 162 (1.6%) were Hispanic. The share of individuals who were Medicaid eligible on the month of diagnosis was 21.3% (174 116 individuals) for the dementia group, 18.4% (36 570 individuals) for the MI group, 17.2% (95 942 individuals) for the COPD group, and 17.7% (8960 individuals) for the colon cancer group. Beneficiaries in the dementia and MI groups had a higher number of chronic conditions than the other 2 groups. The median (IQR) number of months survived following diagnosis in 2016 was 49 (23-49) for COPD, 49 (10-49) for colon cancer, 42 (6-49) for MI, and 37 (11-49) for dementia.

**Table 1.** Characteristics of Older Adults With Diagnoses of Dementia, MI, COPD, and Colon Cancer in 2016^a^

Characteristic	Participants, No. (%) (N = 1 626 127)			
	Dementia (n = 818 862)	MI (n = 199 195)	COPD (n = 557 364)	Colon cancer (n = 50 706)
Age, mean (SD), y	82.0 (7.8)	79.6 (8.0)	77.8 (7.6)	78.3 (7.4)
Gender				
Female	492 146 (60.1)	99 687 (50.0)	303 068 (54.4)	27 293 (53.8)
Male	326 716 (39.9)	99 508 (50.0)	254 296 (45.6)	23 413 (46.2)
Race and ethnicity				
American Indian	4087 (0.5)	1200 (0.6)	2672 (0.5)	266 (0.5)
Asian	13 646 (1.7)	3002 (1.5)	9148 (1.6)	1091 (2.2)
Black	72 935 (8.9)	16 301 (8.2)	44 514 (8.0)	4627 (9.1)
Hispanic	14 063 (1.7)	3081 (1.5)	8272 (1.5)	746 (1.5)
White	714 131 (87.2)	175 611 (88.2)	492 758 (88.4)	43 976 (86.7)
Dual eligible	174 116 (21.3)	36 570 (18.4)	95 942 (17.2)	8960 (17.7)
Chronic Condition Warehouse comorbidities, No.				
0-7	196 752 (24.0)	42 415 (21.3)	189 234 (34.0)	20 034 (39.5)
8-13	353 607 (43.2)	83 460 (41.9)	244 762 (43.9)	19 725 (38.9)
≥14	268 495 (32.8)	73 320 (36.8)	123 364 (22.1)	10 947 (21.6)
Metropolitan county residence	624 935 (76.3)	143 571 (72.1)	416 308 (74.7)	37 680 (74.3)
Social deprivation index				
1-33	289 116 (35.3)	69 168 (34.7)	201 301 (36.1)	18 010 (35.5)
34-66	289 995 (35.4)	73 134 (36.7)	201 057 (36.1)	18 089 (35.7)
67-100	239 742 (29.3)	56 893 (28.6)	155 002 (27.8)	14 607 (28.8)
Time followed, median (IQR), mo	37 (11-49)	42 (6-49)	49 (23-49)	49 (10-49)

Abbreviations: COPD, chronic obstructive pulmonary disease; MI, myocardial infarction.

^a^ The after period includes the month of diagnosis and the following 48 months.

**Figure 1.**
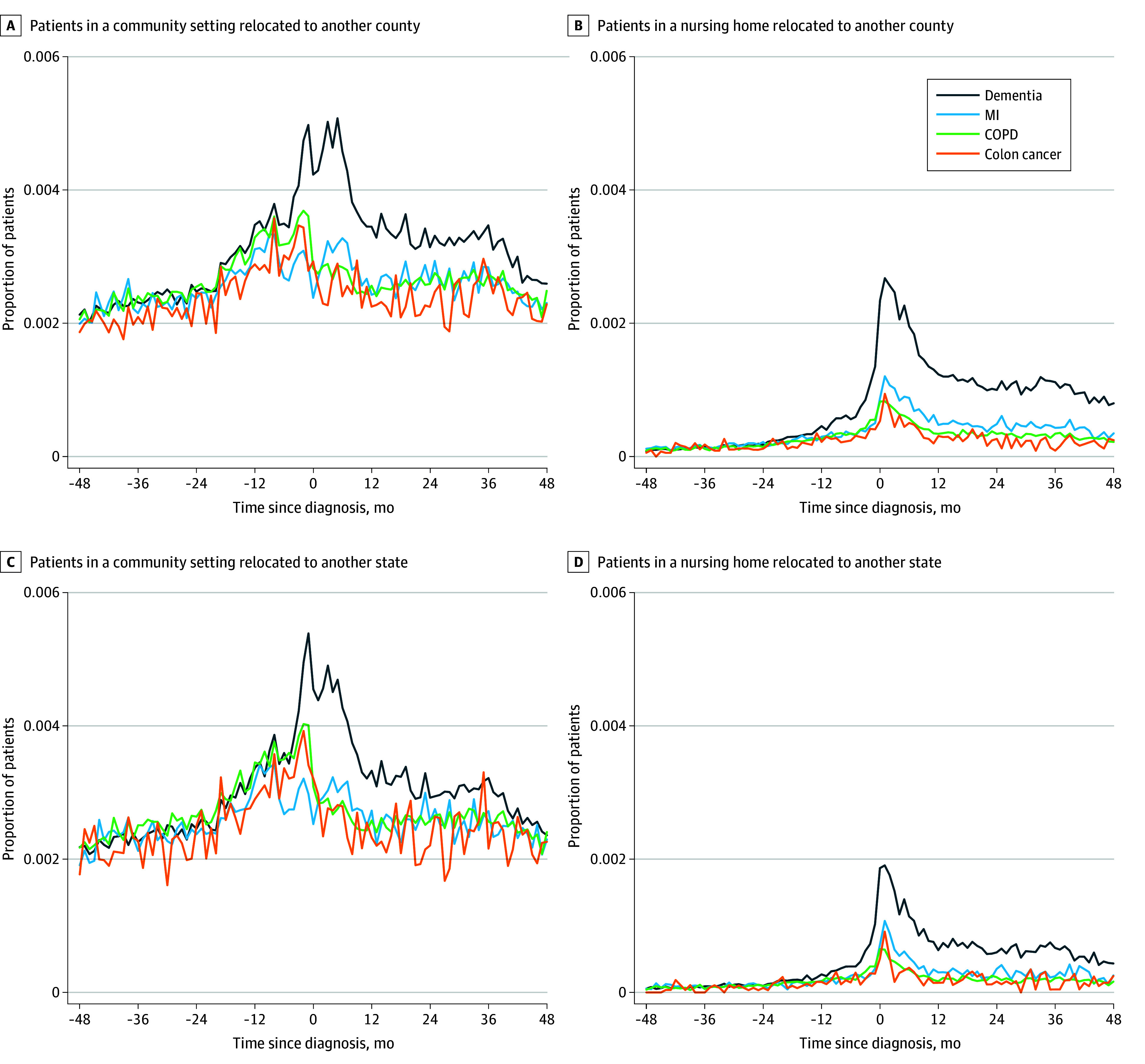
Any Migration Relative to Time of Diagnosis Graphs show proportions of patients in a community setting migrating to another county (A), patients in a nursing home migrating to another county (B), patients in a community setting migrating to another state (C), and patients in a nursing home migrating to another state (D). COPD indicates chronic obstructive pulmonary disease; and MI, myocardial infarction.

Figure 1 shows the rates of intercounty and interstate migration in a community setting and in a nursing over the 8 years before and after the diagnosis date. Across the 4 diagnosis groups, migration rates were similar in the first 3 years (ie, 48 months to 12 months) before diagnosis. Migration rates to nursing home in the dementia group increased substantially around the months immediately before and after diagnosis and remained at a higher level throughout the postdiagnosis period. Migration rates for individuals with MI, COPD, and colon cancer also began to increase before the date of diagnosis but, in time, returned to levels only somewhat higher than during the prediagnosis period. This pattern held for interstate migration as well.

**Figure 2.**
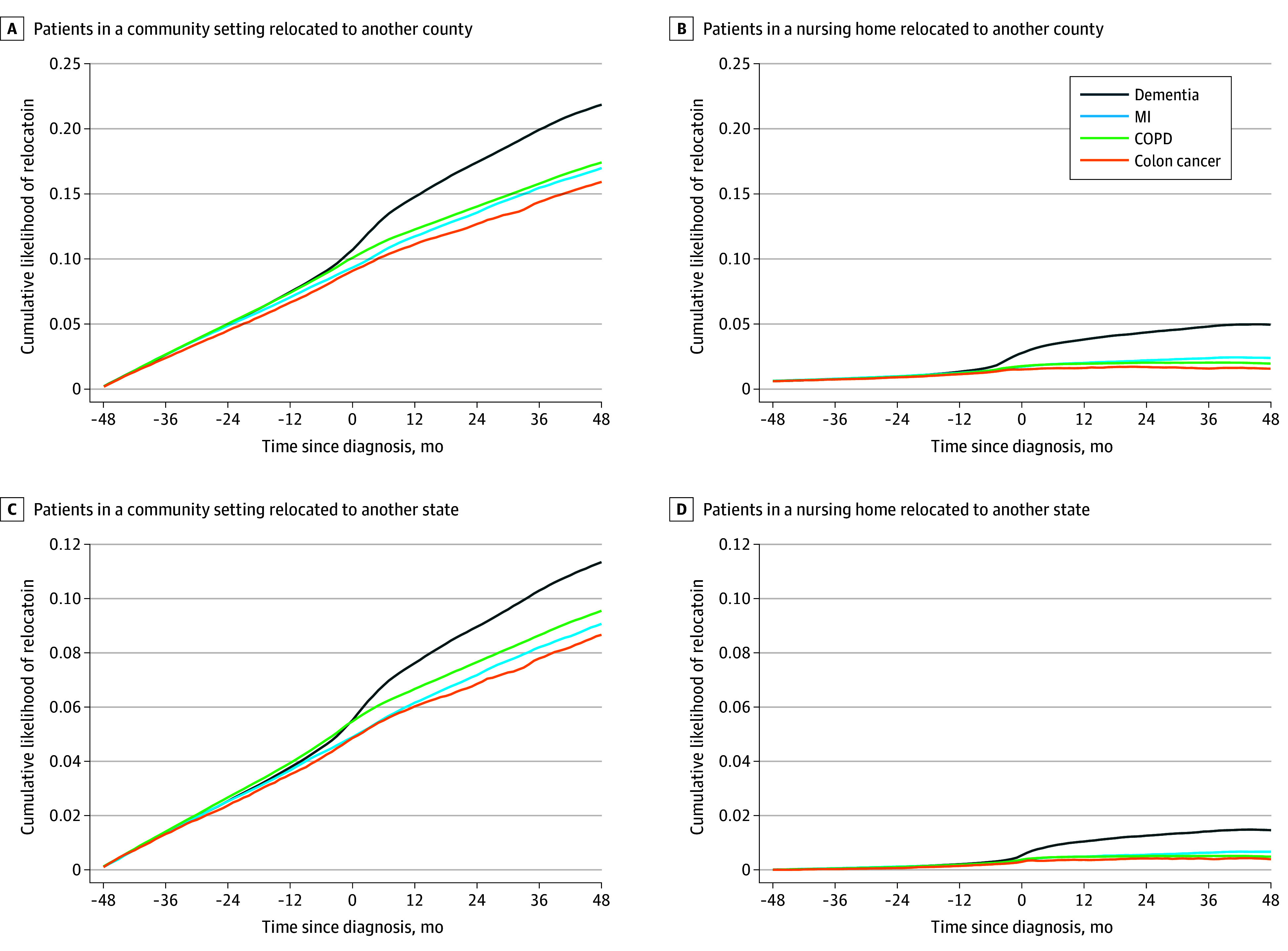
Cumulative Likelihood of Migration Relative to Time of Diagnosis Graphs show cumulative likelihood of migration among patients in a community setting migrating to another county (A), patients in a nursing home migrating to another county (B), patients in a community setting migrating to another state (C), and patients in a nursing home migrating to another state (D). COPD indicates chronic obstructive pulmonary disease; and MI, myocardial infarction.

**Figure 3.**
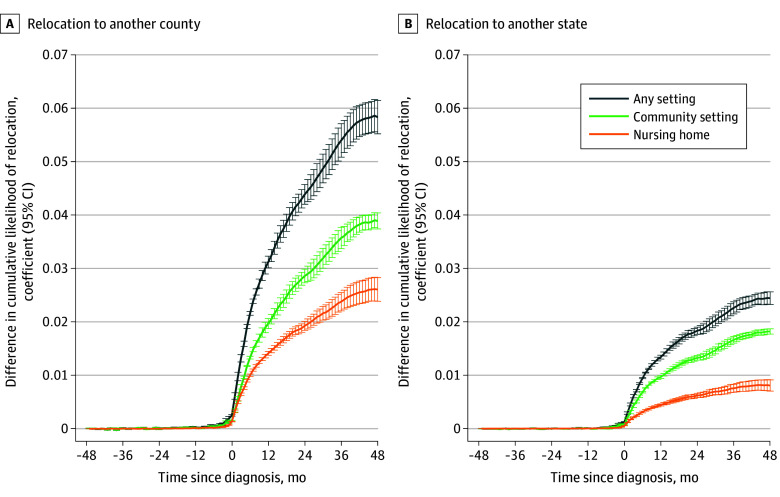
Difference in the Cumulative Likelihood of Migration Between Persons With Dementia and Other Conditions This plot is based on the regression coefficients and associated 95% CIs (error bars) of the dynamic difference in the difference model.

Figure 2 reveals that the cumulative likelihood of migration increased by month for all diagnosis groups at the same rate until the month of diagnosis, after which the cumulative likelihood for persons with dementia increased faster than for the other diagnosis groups. Figure 3 plots the difference in the cumulative likelihood of migration between persons with dementia and other groups by month on the basis of the dynamic difference-in-differences models. The difference in the cumulative likelihood of migration was effectively 0 until the month of diagnosis and continuously increased over the next 48 months. At 48 months after diagnosis, 22% of persons with dementia had moved to a different county, meaning they were 5.8 percentage points (95% CI, 5.5-6.2 percentage points) more likely to experience a migration to another county and 2.4 percentage points (95% CI, 2.3-2.7 percentage points) more likely to experience a migration to another state than those in the other 3 cohorts.

The patterns in cumulative migration can also be seen in the proportion who migrated at any point throughout the 8-year study window (eFigure 1 in Supplement 1). We also observed similar patterns when considering the likelihood of moving to a place 15 miles or further as an outcome (eFigure 2 in Supplement 1). Among those who survived 48 months after diagnosis, compared with persons with the other 3 diagnoses, persons with dementia were approximately 8 percentage points more likely to migrate to a place 15 miles or further. Among those who moved, distances traveled did not vary by diagnosis (eFigure 3 in Supplement 1). Interestingly, migration to a nursing home setting was associated with a shorter distance than for those migrating to a community setting. eFigures 4 and 5 in Supplement 1 demonstrate significant variation in migration rates across states, and eFigure 6 in Supplement 1 highlights that the destination states for individuals with dementia differ substantially from those without dementia.

Table 2 presents the rates of migration for all 4 diagnosis groups in the prediagnosis months, how the rates changed in the postdiagnosis months, and excess migration after diagnosis for persons with dementia compared with the other diagnosis groups. The proportion of individuals with any migration to another county in the prediagnosis period was fairly similar across diagnoses and varied between 9.6% (4857 patients) for colon cancer and 11.4% (93 711 patients) for dementia. In the months following diagnosis, the proportion with any migration increased by 8.5 percentage points (95% CI, 7.6-9.4 percentage points) for persons with dementia, but only by 4.2 to 5.8 percentage points for the other 3 diagnoses. For all diagnoses, we observed a large increase in the likelihood of migration to another county in a nursing home after diagnosis.

**Table 2.** Difference-in-Differences Regression of Outcome Onto Dementia Diagnosis and Postdiagnosis^a^

Diagnosis	Migration to another county			Migration to another state		
	Community	Nursing home	Any	Community	Nursing home	Any
Dementia						
Prediagnosis period^b^	84 891 (10.4)	10 783 (1.3)	93 711 (11.4)	43 578 (5.3)	3627 (0.4)	46 565 (5.7)
Prediagnosis vs postdiagnosis difference^c^	3.4 (2.5 to 4.2)	5.6 (5.1 to 6.1)	8.5 (7.6 to 9.4)	1.0 (0.5 to 1.6)	1.84 (1.5 to 2.0)	2.7 (2.1 to 3.3)
Myocardial infarction						
Prediagnosis period^b^	18 236 (9.2)	1921 (1.0)	19 772 (9.9)	9539 (4.8)	599 (0.3)	10 015 (5.0)
Prediagnosis vs postdiagnosis difference^c^	2.0 (1.1 to 2.8)	4.1 (3.7 to 4.6)	5.8 (4.9 to 6.7)	0.4 (−0.2 to 1.0)	1.3 (1.1 to 1.6)	1.6 (1.0 to 2.3)
Chronic obstructive pulmonary disease						
Prediagnosis period^b^	55 066 (9.9)	5482 (1.0)	59 572 (10.7)	29 828 (5.4)	1867 (0.3)	31 370 (5.6)
Prediagnosis vs postdiagnosis difference^c^	0.9 (0.1 to 1.8)	3.6 (3.1 to 4.1)	4.2 (3.3 to 5.1)	−0.2 (−0.8 to 0.3)	1.1 (0.8 to 1.4)	0.8 (0.2 to 1.4)
Colon cancer						
Prediagnosis period^b^	4498 (8.9)	425 (0.8)	4857 (9.6)	2396 (4.7)	133 (0.3)	2511 (5.0)
Prediagnosis vs postdiagnosis difference^c^	1.6 (0.9 to 2.5)	3.6 (3.1 to 4.1)	4.9 (3.9 to 5.8)	0.2 (−0.4 to 0.8)	1.2 (0.9 to 1.4)	1.3 (0.6 to 1.9)
Difference-in-differences estimate between prediagnosis and postdiagnosis period for dementia vs all others^c^	2.2 (2.0 to 2.3)	1.9 (1.8 to 1.9)	3.9 (3.7 to 4.0)	1.1 (1.0 to 1.2)	0.6 (0.5 to 0.6)	1.6 (1.5 to 1.7)

^a^ These results are based on an analytic dataset where each individual is observed 2 times: prediagnosis and postdiagnosis. Coefficients are based on regressions that include individual fixed effects and number of months observed fixed effects. The 95% CIs are based on robust SEs.

^b^ Data in this row are number (%) of participants.

^c^ Data in this row are percentage points (95% CI).

The last row of Table 2 reveals that excess migration after diagnosis for persons with dementia compared with the other cohorts; relative to the other diagnosis groups, persons with dementia experienced an additional 3.9 percentage point (95% CI, 3.7-4.0 percentage points) higher likelihood of migration to another county after diagnosis. Analyses by destination type found that after diagnosis, persons with dementia experienced a 1.9 percentage point (95% CI, 1.8-1.9 percentage points) higher likelihood of moving to another county in a nursing home and 2.2 percentage point (95% CI, 2.0-2.4 percentage points) higher likelihood of moving to another county in community setting than other persons in other diagnoses groups. Similarly, persons with dementia experienced an additional 1.6 percentage points (95% CI, 1.5-1.7 percentage points) higher likelihood of migration to another state.

eTables 1 and 2 in Supplement 1 exhibit robustness in the estimated excess migration after diagnosis for persons with dementia compared with the other cohorts when estimated separately by demographic characteristics, count of comorbidities, and initial neighborhood characteristics. Of the excess migrations resulting from dementia diagnosis, 55% occurred in community settings, and 45% occurred in institutional settings. Excess county migration due to dementia is approximately 1.3 times higher for female than male individuals (4.2 vs 3.2 percentage points) (eTable 1 in Supplement 1). Dementia-induced migration rates for White and Black beneficiaries were significantly higher than those for other minoritized racial ethnic groups. Excess migration following a dementia diagnosis was approximately 1.7 times higher for non–dual-eligible beneficiaries compared with dual-eligible individuals (4.3 vs 2.6 percentage points). For dual-eligible individuals, the excess migration due to dementia was primarily associated with relocations within nursing home settings. In addition, excess out-of-state migration due to dementia was more than 3 times higher among non–dual-eligible than dual-eligible individuals (1.9 vs 0.6 percentage points). The dementia-induced county migration rate was approximately 1.5 times higher among persons with low comorbidities than persons with high comorbidities (4.5 vs 3.0 percentage points). Dementia-induced migration rates did not vary by much social deprivation index (eTable 2 in Supplement 1). In addition, we found that persons with cognitive difficulties were approximately twice as likely to migrate than persons without such difficulties, with the majority of them migrating to a community setting that is not a group quarter (eTable 3 in Supplement 1). We also noted that approximately 29% of the persons with cognitive difficulties who moved were residing in a household with their offspring (eTable 4 in Supplement 1).

## Discussion

This cohort study revealed that a new dementia diagnosis was significantly associated with residential mobility, leading to a pronounced redistribution across counties and states, compared with diagnoses of MI, COPD, and colon cancer. Our analyses speak to 4 key issues. First, the extent of migration following diagnosis with dementia was considerable, with 22% of individuals moving to a different county within 4 years of diagnosis, a rate 5.8 percentage points (approximately 1.4 times) higher than for those with the other conditions. Second, most migrations occurred within the first year after diagnosis, but they remained higher throughout the entire postdiagnosis period. Third, although transitions to nursing homes after diagnosis were common for all groups, patients with dementia were far more likely to move to other community settings (Table 2). Finally, excess migration postdementia diagnosis varied widely by individual characteristics, including sex, race, and dual eligibility.

This study contributes to 2 related strands of literature at the health-migration nexus. First, most studies examined the association of migration with the spread of infectious disease^10,11,12^ and with health outcomes of migrants.^12,13,14,15,16^ However, very little is known about the impact of a particular disease or diagnosis on migration. This article focuses on the role of a highly prevalent noninfectious chronic condition in leading to internal migration within a high-income country. This can be attributed to the insidious nature of dementia, which gradually erodes patient autonomy, often necessitating migration to environments with additional support and services. Such a shift underscores the profound, often immediate, life changes initiated by a dementia diagnosis, emphasizing its uniqueness compared with other severe health events. Second, the study informs the literature on aging in place.^17^ This literature has traditionally been centered on older adults’ preferences to remain in their home and community as they age and on the adaptations they must make to do so.^18^ Our results show that persons with dementia are more likely than those with other chronic diseases to give up their prior neighborhood and social network in that adaptation process.

Our findings on the heterogeneity of migration patterns have key implications for the literature. Higher dementia-induced migration among female patients may be because older female individuals are more likely to live alone than male individuals, since they live longer and are more likely to be widowed earlier than their male counterparts.^19^ Relatedly, male individuals may also be less capable of spousal caregiving than female individuals. Any migration rate was higher among those with lower comorbidities than those with a medium or high number of comorbidities, regardless of whether the migration was between counties or states. This phenomenon appears consistent with the healthy migrant hypothesis.^20,21^ Lower dementia-induced migration among Hispanic and Asian individuals compared with Black and White older adults may be attributed to the tendency of these groups to more often reside with their offspring.^22^

The variation in dementia-induced migration rates between individuals with and without Medicaid enrollment carries important policy implications. For those without dual eligibility, most dementia-related migration occurs within community settings. In contrast, migration is predominantly within nursing homes for individuals with dual eligibility. Furthermore, the rate of excess interstate migration is much lower for those with dual eligibility than their counterpart. These findings suggest that Medicaid eligibility plays a critical role in aging in place for persons with dementia. Furthermore, eFigures 4 and 5 in Supplement 1 demonstrate significant variation in migration rates across states, whereas eFigure 6 in Supplement 1 highlights that the destination states for individuals with dementia differ substantially from those without dementia, likely influenced by differences in long-term care policies and generosity of state Medicaid policies across states.

### Limitations

This study has limitations that should be mentioned. Although this study demonstrated the association between dementia and migration, disentangling the reasons for the observed migration patterns is beyond this study’s scope. One possible explanation is that individuals with dementia and their caregivers may choose to move closer to family or informal caregivers, either with independent housing arrangements or entering formal long-term care services such as assisted living communities and nursing homes. A simple analysis of older adults in the American Community Survey suggests that persons with cognitive difficulties are approximately 2 times more likely to migrate than persons without such difficulties, with the majority of them migrating to a community setting that is not a group quarter (eTable 3 in Supplement 1). We also noted that approximately 29% of the persons with cognitive difficulties who moved were residing in a household with their offspring (eTable 4 in Supplement 1). Another limitation of our analysis is that our data do not tell the degree of dementia severity at the time of diagnosis. We could not determine whether these effects are similar among Medicare Advantage participants, who may differ from the fee-for-service population in ways that might affect migration. We also could not estimate a base migration rate for the Medicare population without the specified conditions.

## Conclusions

Our findings illuminate the profound impact that a dementia diagnosis can have on an individual’s residential choices, challenging traditional conceptions of aging in place and adding depth to our understanding of the interplay between health and migration. As the global population ages and dementia prevalence increases, it becomes crucial for policymakers and communities to recognize and address the unique migration patterns and needs of individuals with this life-altering condition.

## Notes

Supplement 1. **eTable 1.** Difference in difference regression of outcome onto ADRD diagnosis and post-diagnosis (in percentage points) among different sub-groups based on beneficiary characteristics **eTable 2.** Difference in difference regression of outcome onto ADRD diagnosis and post-diagnosis (in percentage points) among different sub-groups based on beneficiary zip code social deprivation index and county rurality **eFigure 1.** Proportion migrated to another county in a community and nursing home setting in 8 years as a function of the months survived **eFigure 2.** Proportion of individuals migrated to a place 15 miles or farther away from the first observed zip code **eFigure 3.** Box plot of distances moved among those who migrated to a different county **eFigure 4.** Share of persons with dementia migrated to a different county in four years post-diagnosis in community and nursing home setting by origin state **eFigure 5.** Share migrated to a different state in four years post-diagnosis in any setting by origin state for persons with dementia and other diagnoses **eFigure 6.** Geographic distribution of destination states among persons with dementia who moved to another state relative to those with other diagnoses **eTable 3.** Proportion migrated in last one year among Americans with age 65 and over, based on American Community Survey 2019 **eTable 4.** Proportion living in a household with own offspring among Americans with age 65 and over and residing in a community setting, based on American Community Survey 2019

Supplement 2.
Data Sharing Statement
