# Supplementary material for: Patterns of Migration Following Dementia Diagnosis
Source: JAMA Netw Open. 2024 Oct 14;7(10):e2439499. doi: 10.1001/jamanetworkopen.2024.39499 (PMC11474419; doi:10.1001/jamanetworkopen.2024.39499)
Supplement: Supplement 1. — eTable 1. Difference in difference regression of outcome onto ADRD diagnosis and post-diagnosis (in percentage points) among different sub-groups based on beneficiary characteristics eTable 2. Difference in difference regression of outcome onto ADRD diagnosis and post-diagnosis (in percentage points) among different sub-groups based on beneficiary zip code social deprivation index and county rurality eFigure 1. Proportion migrated to another county in a community and nursing home setting in 8 years as a function of the months survived eFigure 2. Proportion of individuals migrated to a place 15 miles or farther away from the first observed zip code eFigure 3. Box plot of distances moved among those who migrated to a different county eFigure 4. Share of persons with dementia migrated to a different county in four years post-diagnosis in community and nursing home setting by origin state eFigure 5. Share migrated to a different state in four years post-diagnosis in any setting by origin state for persons with dementia and other diagnoses eFigure 6. Geographic distribution of destination states among persons with dementia who moved to another state relative to those with other diagnoses eTable 3. Proportion migrated in last one year among Americans with age 65 and over, based on American Community Survey 2019 eTable 4. Proportion living in a household with own offspring among Americans with age 65 and over and residing in a community setting, based on American Community Survey 2019 [file jamanetwopen-e2439499-s001.pdf]

Supplemental Online Content

Rahman M, Thapa BB, Santostefano C, et al. Patterns of migration following dementia diagnosis. *JAMA Netw Open*. 2024;7(10):e2439499.
doi:10.1001/jamanetworkopen.2024.39499

eTable 1. Difference in difference regression of outcome onto ADRD diagnosis and post-diagnosis (in percentage points) among different sub-groups based on beneficiary characteristics
eTable 2. Difference in difference regression of outcome onto ADRD diagnosis and post-diagnosis (in percentage points) among different sub-groups based on beneficiary zip code social deprivation index and county rurality
eFigure 1. Proportion migrated to another county in a community and nursing home setting in 8 years as a function of the months survived
eFigure 2. Proportion of individuals migrated to a place 15 miles or farther away from the first observed zip code
eFigure 3. Box plot of distances moved among those who migrated to a different county
eFigure 4. Share of persons with dementia migrated to a different county in four years post-diagnosis in community and nursing home setting by origin state
eFigure 5. Share migrated to a different state in four years post-diagnosis in any setting by origin state for persons with dementia and other diagnoses
eFigure 6. Geographic distribution of destination states among persons with dementia who moved to another state relative to those with other diagnoses
eTable 3. Proportion migrated in last one year among Americans with age 65 and over, based on American Community Survey 2019
eTable 4. Proportion living in a household with own offspring among Americans with age 65 and over and residing in a community setting, based on American Community Survey 2019

This supplemental material has been provided by the authors to give readers additional information about their work.

© 2024 Rahman M et al. *JAMA Network Open.*

eTable 1: Difference in difference regression of outcome onto ADRD diagnosis and post-diagnosis (in percentage points) among different sub-groups based on beneficiary characteristics

	Migration to another county			Migration to another state		
	Community	Nursing home	Any	Community	Nursing home	Any
Entire sample	2.156***	1.874***	3.865***	1.090***	0.587***	1.633***
N=3,252,230	[2.036 - 2.277]	[1.814 - 1.934]	[3.735 - 3.995]	[1.002 - 1.178]	[0.552 - 0.622]	[1.540 - 1.725]
Male	1.774***	1.606***	3.232***	0.944***	0.474***	1.380***
N=1,407,842	[1.595 - 1.953]	[1.521 - 1.690]	[3.041 - 3.423]	[0.812 - 1.075]	[0.425 - 0.523]	[1.242 - 1.518]
Female	2.418***	2.032***	4.268***	1.191***	0.658***	1.799***
N=1,844,388	[2.254 - 2.581]	[1.949 - 2.116]	[4.091 - 4.445]	[1.072 - 1.310]	[0.609 - 0.706]	[1.673 - 1.925]
White	2.278***	1.859***	3.981***	1.147***	0.588***	1.695***
N=2,852,928	[2.147 - 2.409]	[1.795 - 1.923]	[3.841 - 4.122]	[1.052 - 1.242]	[0.551 - 0.625]	[1.595 - 1.796]
Black	1.814***	2.212***	3.779***	0.886***	0.682***	1.496***
N=276,754	[1.445 - 2.182]	[1.989 - 2.435]	[3.372 - 4.187]	[0.604 - 1.168]	[0.548 - 0.815]	[1.195 - 1.797]
Other race	0.743***	1.464***	2.021***	0.482**	0.350***	0.751***
N=122,548	[0.180 - 1.307]	[1.217 - 1.712]	[1.426 - 2.616]	[0.0824 - 0.881]	[0.213 - 0.488]	[0.338 - 1.165]
Not dual eligible	2.842***	1.590***	4.275***	1.443***	0.555***	1.952***
N=2,621,054	[2.705 - 2.980]	[1.534 - 1.646]	[4.131 - 4.420]	[1.342 - 1.545]	[0.520 - 0.590]	[1.846 - 2.057]
Dual eligible	0.0282	2.803***	2.629***	-0.0276	0.705***	0.638***
N=631,176	[-0.223 - 0.280]	[2.604 - 3.002]	[2.329 - 2.928]	[-0.200 - 0.144]	[0.600 - 0.810]	[0.446 - 0.831]
Low comorbidities	2.469***	2.182***	4.513***	1.292***	0.658***	1.919***
N= 896,870	[2.224 - 2.713]	[2.069 - 2.295]	[4.253 - 4.774]	[1.111 - 1.473]	[0.593 - 0.723]	[1.730 - 2.108]
Medium comorbidities	2.500***	1.734***	4.062***	1.294***	0.549***	1.805***
N=1,403,108	[2.317 - 2.683]	[1.650 - 1.818]	[3.867 - 4.258]	[1.160 - 1.427]	[0.499 - 0.598]	[1.665 - 1.945]
High comorbidities	1.467***	1.723***	3.015***	0.658***	0.556***	1.149***
N=952,252	[1.251 - 1.684]	[1.597 - 1.849]	[2.776 - 3.254]	[0.502 - 0.814]	[0.483 - 0.629]	[0.981 - 1.316]

© 2024 Rahman M et al. *JAMA Network Open.*

eTable 2: Difference in difference regression of outcome onto ADRD diagnosis and post-diagnosis (in percentage points) among different sub-groups based on beneficiary zip code social deprivation index and county rurality

	Migration to another county			Migration to another state		
	Community	Nursing home	Any	Community	Nursing home	Any
Entire sample	2.156***	1.874***	3.865***	1.090***	0.587***	1.633***
N=3,252,230	[2.036 - 2.277]	[1.814 - 1.934]	[3.735 - 3.995]	[1.002 - 1.178]	[0.552 - 0.622]	[1.540 - 1.725]
SDI: 0-33	2.721***	1.671***	4.233***	1.470***	0.590***	2.005***
N=1,136,206	[2.507 - 2.934]	[1.575 - 1.767]	[4.006 - 4.461]	[1.311 - 1.629]	[0.532 - 0.648]	[1.838 - 2.171]
SDI: 34-66	2.056***	1.935***	3.832***	1.016***	0.601***	1.580***
N=1,145,412	[1.852 - 2.260]	[1.833 - 2.038]	[3.612 - 4.051]	[0.869 - 1.163]	[0.542 - 0.660]	[1.425 - 1.735]
SDI: 67-100	1.673***	2.024***	3.520***	0.779***	0.565***	1.303***
N=917,168	[1.460 - 1.887]	[1.907 - 2.141]	[3.287 - 3.753]	[0.626 - 0.932]	[0.499 - 0.631]	[1.141 - 1.466]
Metropolitan counties	2.362***	1.743***	3.950***	1.240***	0.559***	1.755***
N=2,444,988	[2.222 - 2.501]	[1.676 - 1.810]	[3.800 - 4.099]	[1.137 - 1.344]	[0.519 - 0.599]	[1.647 - 1.863]
Non Metro counties	1.578***	2.327***	3.702***	0.672***	0.683***	1.311***
N=807,242	[1.337 - 1.820]	[2.195 - 2.458]	[3.438 - 3.966]	[0.503 - 0.841]	[0.611 - 0.756]	[1.131 - 1.490]

eFigure 1: Proportion migrated to another county in a community and nursing home setting in 8 years as a function of the months survived

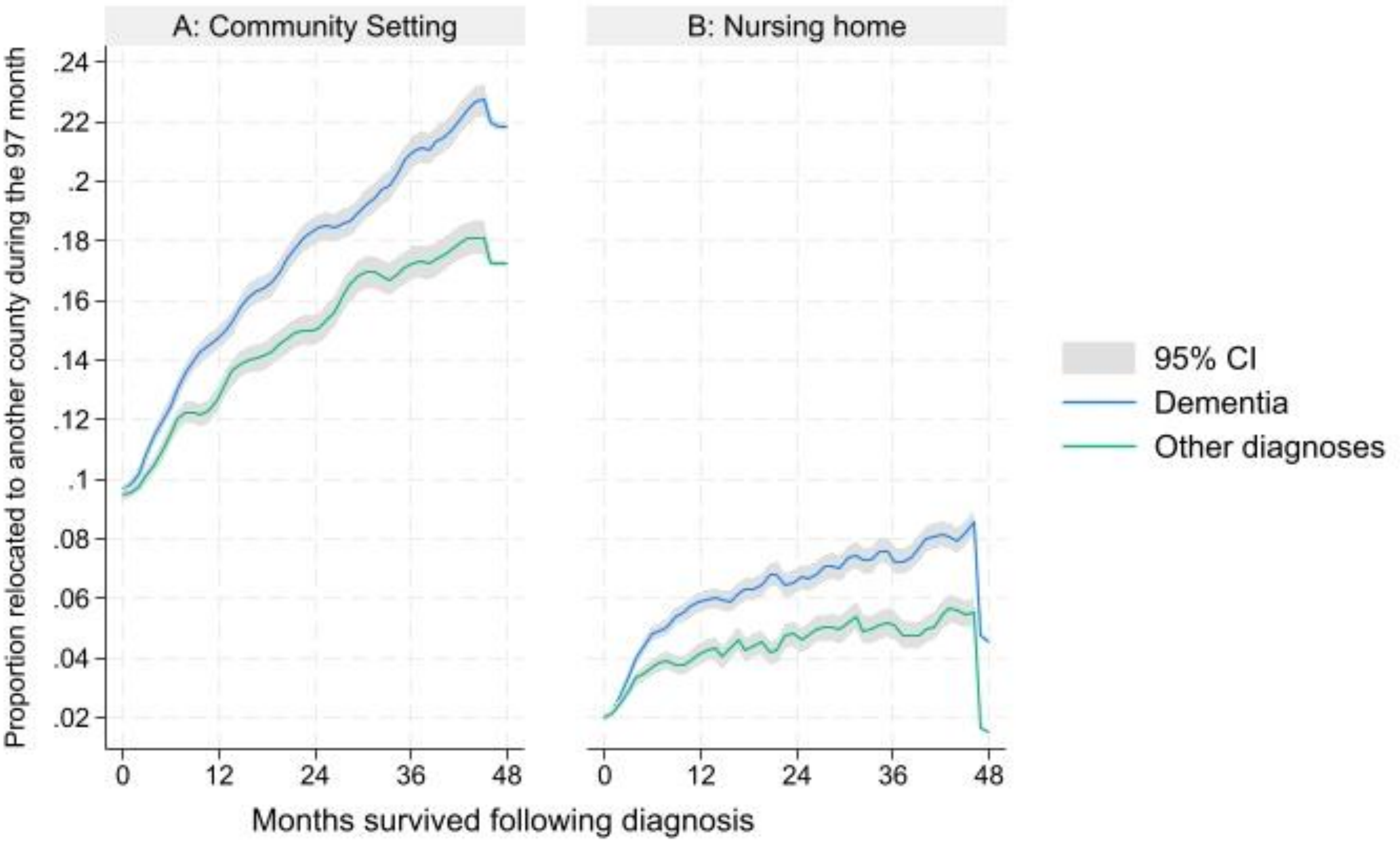

Note: This graph is based on individual level data. We used polynomial smoothing plot (twoway lpolyci command in stata) of any migration in the entire 8 years of follow up (a binary variable) onto number of months survived after diagnosis (0 the month of diagnosis, capped at 48).

© 2024 Rahman M et al. *JAMA Network Open.*

eFigure 2: Proportion of individuals migrated to a place 15 miles or farther away from the first observed zip code

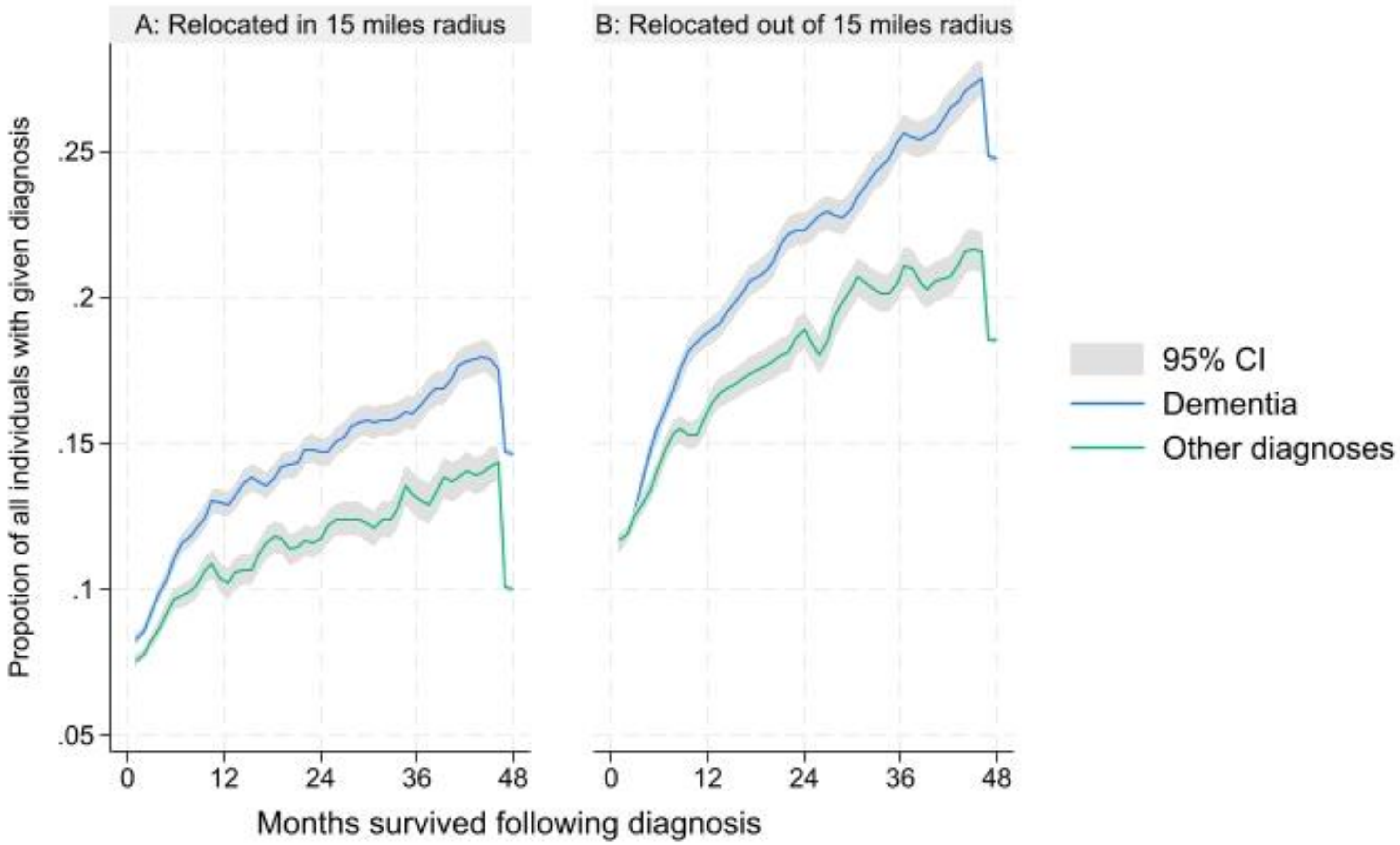

Note: This graph is based on individual-level data. We created two binary outcome variables: any relocation within a 15-mile radius and outside the 15-mile radius from the first zip code observed. We then used a polynomial smoothing plot of these outcomes onto a number of months survived after diagnosis (0 the month of diagnosis, capped at 48).

© 2024 Rahman M et al. *JAMA Network Open.*

eFigure 3: Box plot of distances moved among those who migrated to a different county

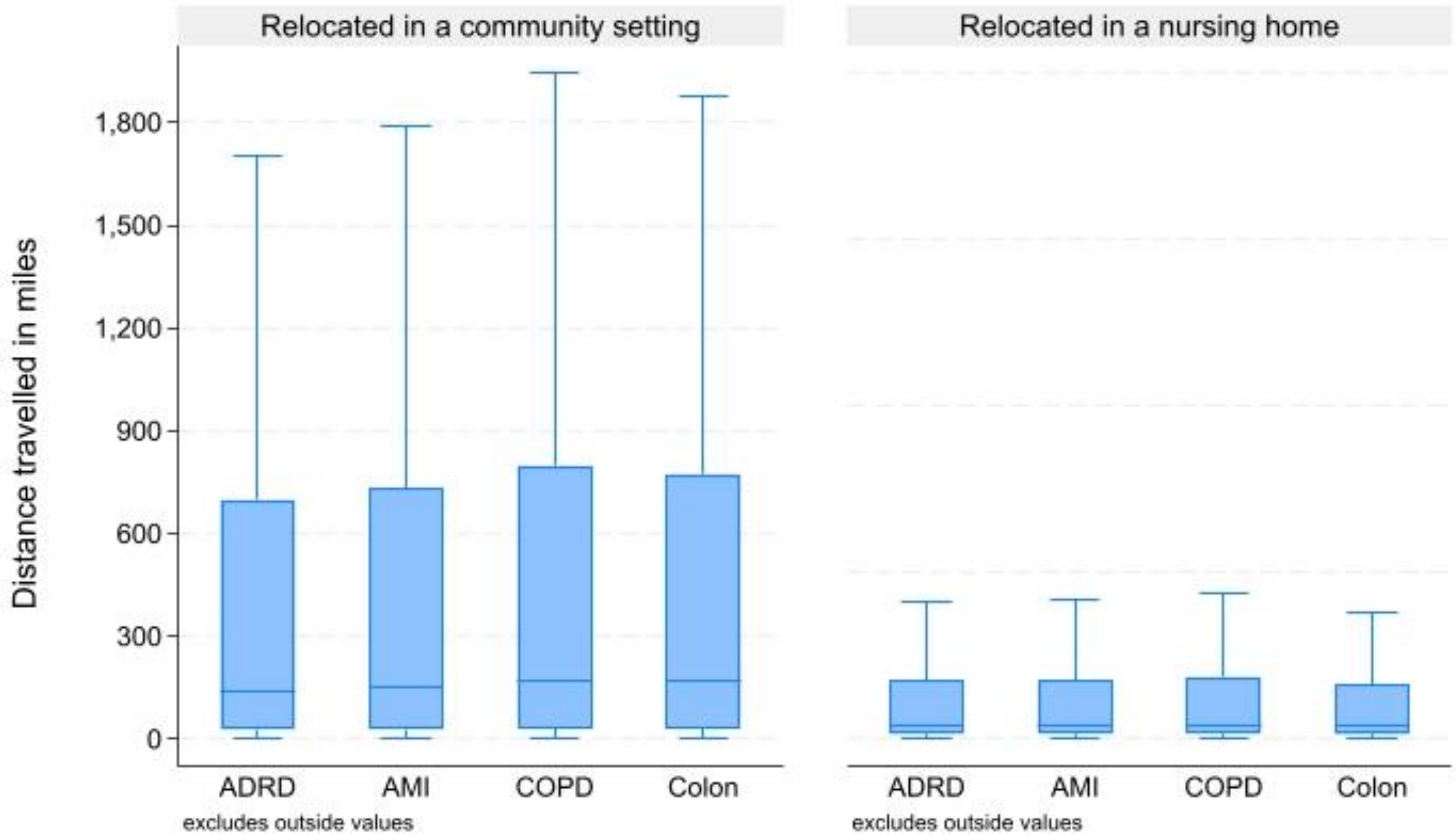

© 2024 Rahman M et al. *JAMA Network Open.*

eFigure 4: Share of persons with dementia migrated to a different county in four years post-diagnosis in community and nursing home setting by origin state

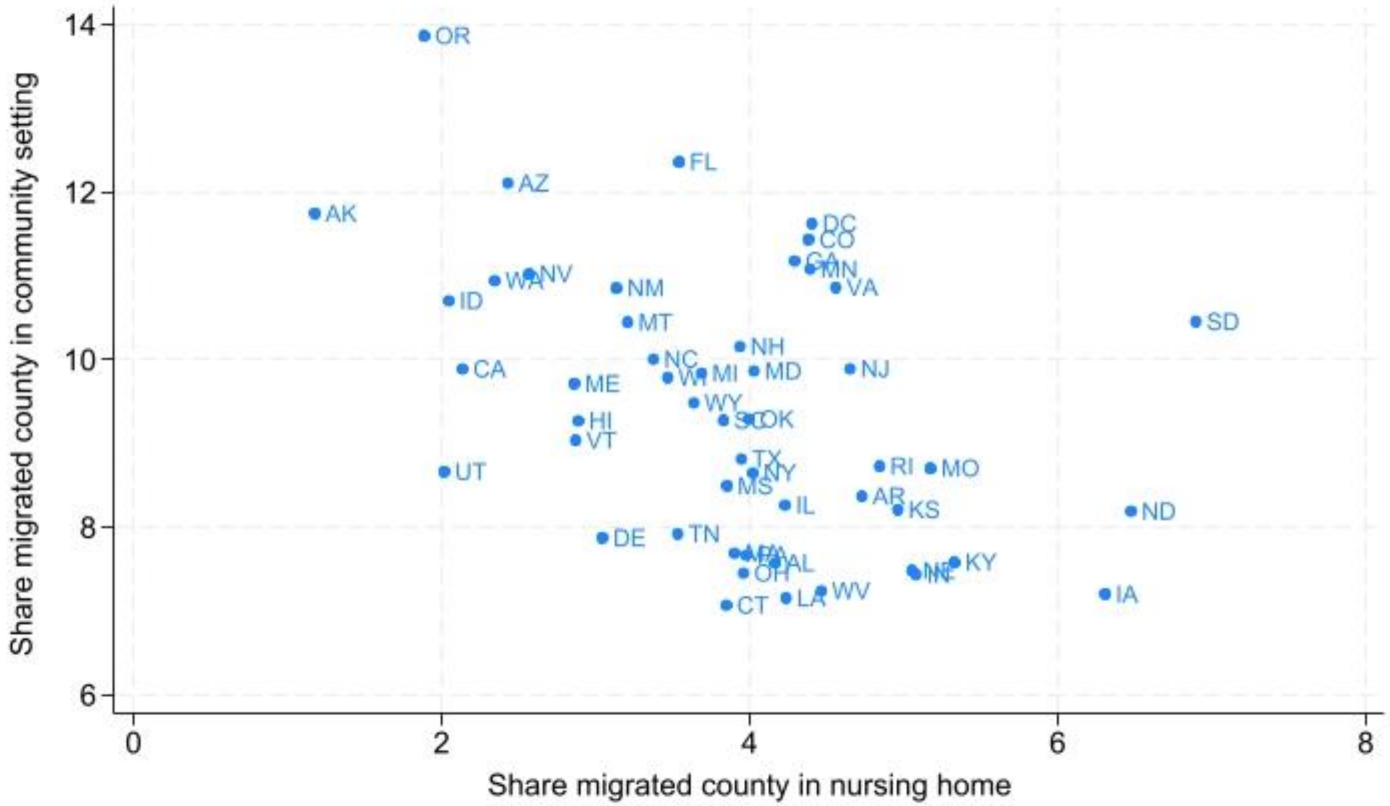

Note: We used individual-level data for persons with dementia for the post-period, regressed the two outcomes on state fixed effects and individual characteristics, and estimated the margins for the state fixed effects. We plotted the margins for the two outcomes using a scatter plot.

© 2024 Rahman M et al. *JAMA Network Open.*

eFigure 5: Share migrated to a different state in four years post-diagnosis in any setting by origin state for persons with dementia and other diagnoses

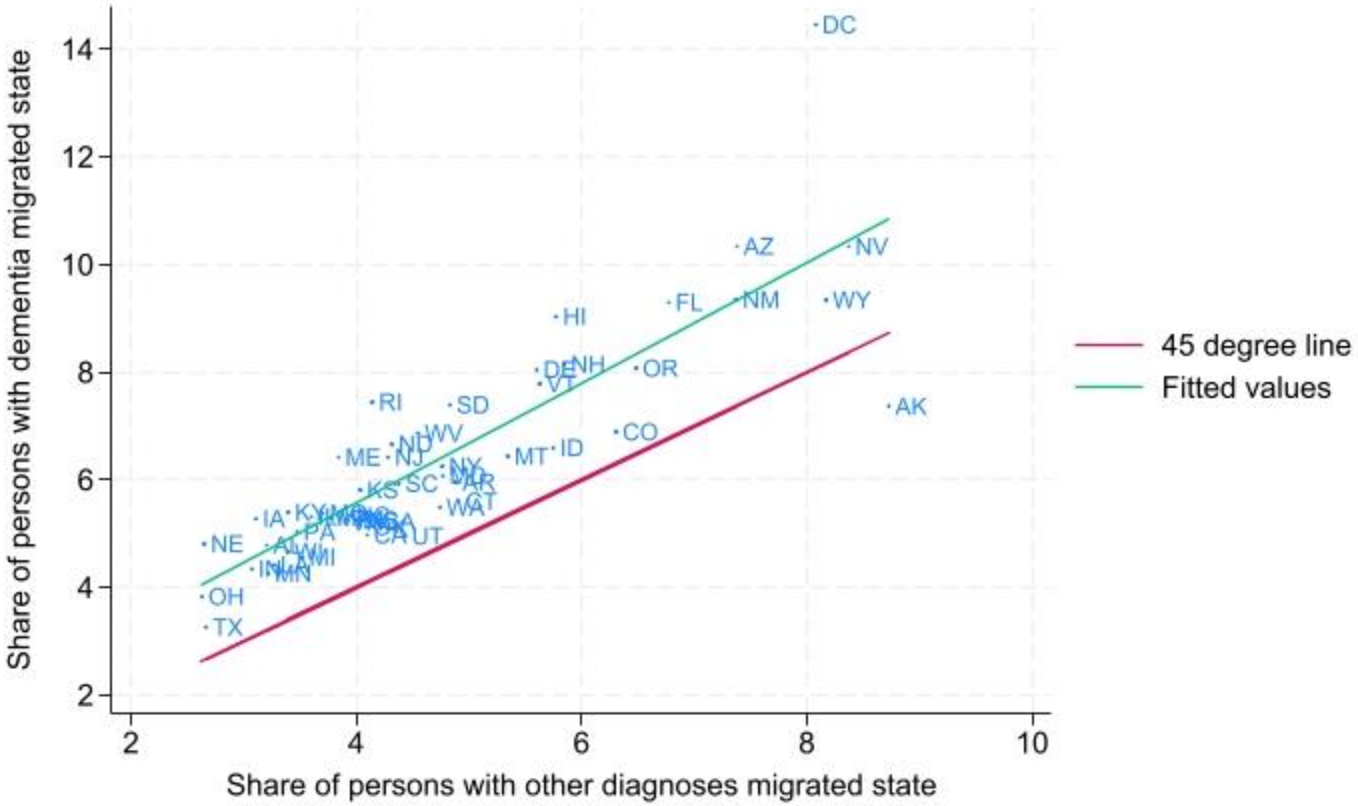

Note: We used individual-level data for the post-period, regressed the any out-of-state migration on state fixed effects and individual characteristics separately for persons with and without dementia, and estimated the margins for the state fixed effects. We plotted the margins for the two groups using a scatter plot.

© 2024 Rahman M et al. *JAMA Network Open.*

eFigure 6: Geographic distribution of destination states among persons with dementia who moved to another state relative to those with other diagnoses

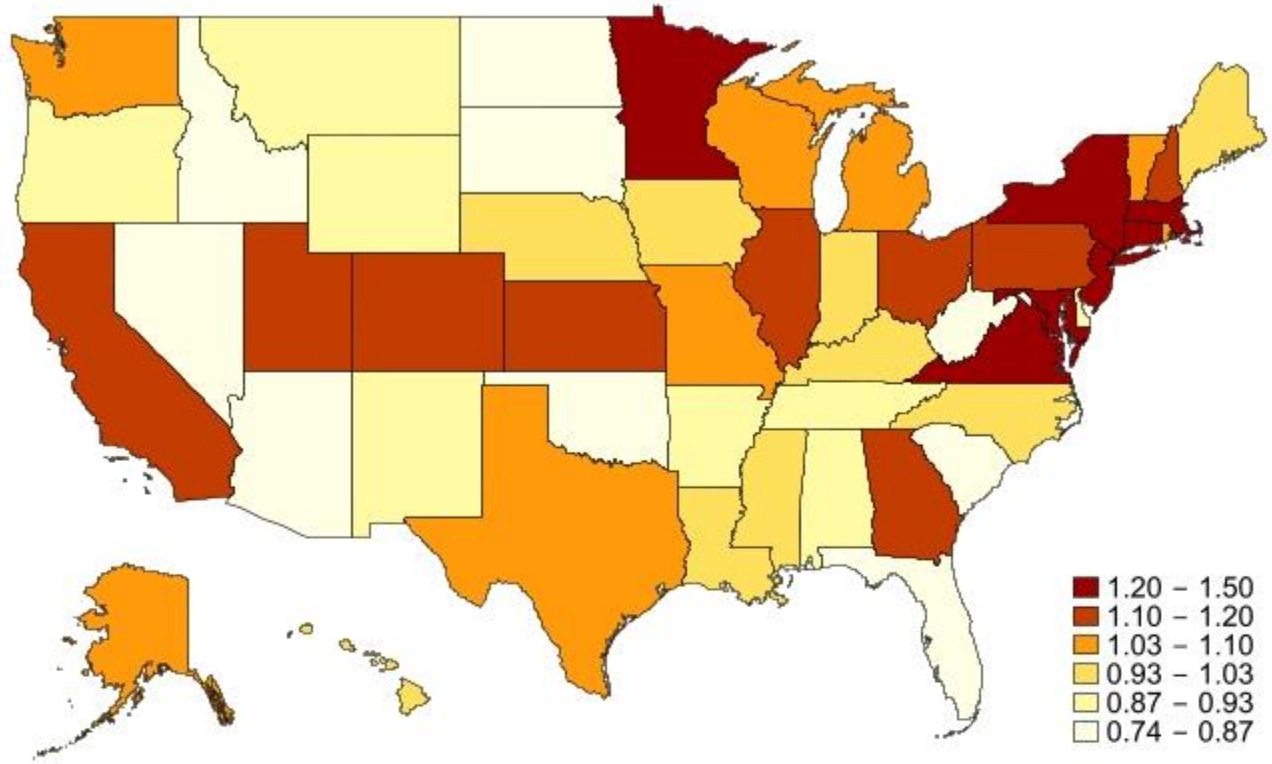

Note: We counted the number of out-of-state migrants by the destination states for persons with and without dementia. We then calculated the proportion them going to a destination state. We calculated the ratio of the proportions for persons with and without dementia and mapped the ratios.

© 2024 Rahman M et al. *JAMA Network Open.*

eTable 3: Proportion migrated in last one year among Americans with age 65 and over, based on American Community Survey 2019

	Percentage of population moved to a community setting	Percent of population moved to a group quarter
Without cognitive difficulties	5.16%	0.34%
With cognitive difficulties	7.16%	3.78%

eTable 4: Proportion living in a household with own offspring among Americans with age 65 and over and residing in a community setting, based on American Community Survey 2019

	Who did not migrate in last one year	Who migrated in last one year
Without cognitive difficulties	17.39%	18.06%
With cognitive difficulties	27.76%	29.19%

© 2024 Rahman M et al. *JAMA Network Open.*
