# Supplementary material for: Patterns of Migration Following Dementia Diagnosis
Source: JAMA Netw Open. 2024 Oct 14;7(10):e2439499. doi: 10.1001/jamanetworkopen.2024.39499 (PMC11474419; doi:10.1001/jamanetworkopen.2024.39499)
Supplement: Supplement 2. — Data Sharing Statement [file jamanetwopen-e2439499-s002.pdf]

Data Sharing Statement

Rahman. Patterns of Migration Following Dementia Diagnosis. *JAMA Netw Open*. Published October 14, 2024. doi:10.1001/jamanetworkopen.2024.39499

Data
Data available: No
Additional Information:
We did not generate any new primary data for this project. We created analytic files based on the existing secondary data to conduct our analysis. While we cannot make these secondary analytic files available due to the DUA requirements, we will make all code used to generate our files publicly available. The data dictionaries, data retrieval documents (including variable names), and statistical analysis plans for this manuscript can be found at https://doi.org/10.26300/crq0-ge81. We included SAS codes to build the analytic file using RESDAC-provided data and the Stata codes used to analyze the analytic file to obtain the results for this manuscript.
